# Supplementary material for: The relationship between workplace bullying and family functioning: A systematic review
Source: PLoS One. 2024 Sep 17;19(9):e0310300. doi: 10.1371/journal.pone.0310300 (PMC11407676; doi:10.1371/journal.pone.0310300)
Supplement: S1 Table — (DOCX) [file pone.0310300.s002.docx]

# Search terms and strategies for four databases

**Keywords used for literature searches.**

| Group 1 | Group 2 | Group 3 |
| --- | --- | --- |
| Work* | Bullying | Family function* |
| Job | Mobbing | Family adaptability |
| Occupational | Harassment | Family relation* |
| Employee  Worker | Mistreatment  Victimization | Family cohesion  Family communication |
|  | Ostracism | Family interaction |
|  | Exclusion  Emotional abuse  Psychological aggression  Abusive supervision | Family health  Family dysfunction  Family conflict  Family disruption |
|  |  | Family undermining |

**Search strings used for the literature search in each database.**

| **Scopus-keywords** |
| --- |
| ( ( TITLE-ABS-KEY ( **work*** ) OR TITLE-ABS-KEY ( **job** ) OR TITLE-ABS-KEY ( **occupational** ) OR TITLE-ABS-KEY ( **employee** ) OR TITLE-ABS-KEY ( **worker )** ) ) AND ( ( TITLE-ABS-KEY ( **bullying** ) OR TITLE-ABS-KEY ( **mobbing** ) OR TITLE-ABS-KEY ( **harassment** ) OR TITLE-ABS-KEY ( **mistreatment** ) OR TITLE-ABS-KEY ( **victimization** ) OR TITLE-ABS-KEY ( **ostracism** ) OR TITLE-ABS-KEY ( **exclusion** ) OR TITLE-ABS-KEY ( **"emotional abuse"** ) OR TITLE-ABS-KEY (**"psychological aggression"**) OR TITLE-ABS-KEY (**"abusive supervision"** ) ) ) AND ( ( TITLE-ABS-KEY ( **"family function*"** ) OR TITLE-ABS-KEY ( **"family adaptability"** ) OR TITLE-ABS-KEY ( **"family relation*"** ) OR TITLE-ABS-KEY ( **"family cohesion"** ) OR TITLE-ABS-KEY ( **"family communication"** ) OR TITLE-ABS-KEY ( **"family interactions"** ) OR TITLE-ABS-KEY ( **"family health"** ) OR TITLE-ABS-KEY ( **"family dysfunction"** ) OR TITLE-ABS-KEY ( **"family conflict"** ) OR TITLE-ABS-KEY ( **"family disruption"** ) OR TITLE-ABS-KEY ( **"family undermining"** ) ) ) AND ( LIMIT-TO ( DOCTYPE, "ar" ) ) AND ( LIMIT-TO ( LANGUAGE, "English" ) ) |
| **Web of Science-keywords** |
| **Query #1**  **TS= (work*** or **job** or **occupational** or **employee** or **worker)**  **Query #2**  **TS= (bullying** or **mobbing** or **harassment** or **mistreatment** or **victimization** or **ostracism** or  **exclusion** or **"emotional abuse"** or **"psychological aggression"** or **"abusive supervision")**  **Query #3**  **TS= ("family function*"** or **"family adaptability"** or **"family relation*"** or **"family cohesion"** or **"family communication"** or **"family interactions"** or **"family health"** or **"family dysfunction"** or **"family conflict"** or **"family disruption"** or **"family undermining")**  **Query #4**  **#1 AND #2 AND #3** and **Article** (Document Types) and **English** (Languages) |

| **PsycINFO-keywords** |
| --- |
| **S1:** SU **work*** OR **job** OR **occupational** OR **employee** OR **worker**  **S2:** SU **bullying** OR **mobbing** OR **harassment** OR **mistreatment** OR **victimization** OR **ostracism** OR **exclusion** OR **“emotional abuse”** OR **“psychological aggression”** OR **“abusive supervision”**  **S3:** SU **“family function*”** OR **“family adaptability”** OR **“family relation*”** OR **“family cohesion”** OR **“family communication”** OR **“family interactions”** OR **“family health”** OR **“family dysfunction”** OR **“family conflict”** OR **“family disruption”** OR **“family undermining”**  **S4: S1 AND S2 AND S3**  Limiters-Publication Type: All Journals; English language |
| **PubMed-keywords** |
| **((((((work*) OR (job)) OR (occupational)) OR (employee)) OR (worker)) AND (((((((((((((family[MeSH Terms]) OR ("family relations/psychology"[MeSH Terms])) OR ("family conflict/psychology"[MeSH Terms])) OR ("family support"[MeSH Terms])) OR ("family health"[MeSH Terms])) OR ("family function*")) OR ("family adaptability")) OR ("family cohesion")) OR ("family communication")) OR ("family interaction")) OR ("family dysfunction")) OR ("family disruption")) OR ("family undermining"))) AND ((((((((((("bullying/psychology"[MeSH Terms]) OR ("aggression/psychology"[MeSH Terms])) OR ("emotional abuse/psychology"[MeSH Terms])) OR ("harassment, non sexual"[MeSH Terms])) OR ("ostracism"[MeSH Terms])) OR (mobbing)) OR (victimization)) OR (mistreatment)) OR (exclusion)) OR (undermining)) OR ("abusive supervision"))**  **Filters:** **Associated data, English** |
