# Supplementary material for: The relationship between workplace bullying and family functioning: A systematic review
Source: PLoS One. 2024 Sep 17;19(9):e0310300. doi: 10.1371/journal.pone.0310300 (PMC11407676; doi:10.1371/journal.pone.0310300)
Supplement: S2 Table — (DOCX) [file pone.0310300.s003.docx]

# S3 Table. Revised checklist for methodological quality.

| 1 | Sampling method | - Non-probability sampling: purposive, quota, convenience, and snowball sampling - 0. - Probability sampling: simple random, systematic, stratified, cluster, two-stage, and multi-stage sampling - 1. |
| --- | --- | --- |
| 2 | **Was the response rate reported?** | - **Not reported - 0.** - **Response rate below 50% - 0.** - **Response rate at 50% or above - 1.** |
| 3 | **Are the individuals selected to participate in the study likely to be representative of the target population?** | - **No - 0.** - **Yes - 1.** |
| 4 | **Is there a risk of selection bias caused by the inadequate selection of participants?** | - **High risk - 0.** - **Low risk - 1.** |
| 5 | **How was workplace bullying measured?** | - **Self-labeling without definition of bullying concept - 0.** - **Self-labeling with a definition of bullying concept - 1.** - **Behavioral checklist (e.g., NAQ, LIPT) - 1.** |
| 6 | **How was family functioning assessed?** | - **Self-report - 0.** - **Data from spouses or family members - 1.** |
| 7 | **Is there a risk of bias caused by the inadequate measurement of exposure?** | - **High risk - 0.** - **Low risk - 1.** |
| 8 | **Are the statistical methods appropriate for the study design?** | - **No/Cannot tell - 0.** - **Yes - 1.** |
| 9 | **Were meaningful demographic covariates included?** | - **No - 0.** - **Yes - 1.** |
| 10 | **Is there a risk of bias caused by the inadequate confirmation and consideration of confounding variables?** | - **High risk - 0.** - **Low risk - 1.** |
