# Supplementary material for: The relationship between workplace bullying and family functioning: A systematic review
Source: PLoS One. 2024 Sep 17;19(9):e0310300. doi: 10.1371/journal.pone.0310300 (PMC11407676; doi:10.1371/journal.pone.0310300)
Supplement: Attachment — Submitted filename: Response to Reviewers.docx [file pone.0310300.s006.docx]

**RESPONSE TO EDITOR AND REVIEWERS’ COMMENTS**

Dear Editors,

We would like to begin with our sincere appreciation for all the valuable comments, insightful suggestions, and thoughtful corrections offered by the reviewers and editors to our manuscript. The comments and suggestions helped us to improve the quality of the manuscript. We have revised the manuscript, highlighting major changes in blue. These changes are summarized below, following a point-by-point response to the reviewer’s comments.

**REVIEWER 1**

#Introduction
Although the introduction provides a comprehensive overview of the research problem and provide statistics from the global perspective on workplace bullying but some of the information is repeated and the introduction can be made concise. I believe authors can provide more specific examples of existing research gaps by citing more work, this would strengthen the importance of the research. I would suggest research questions and hypotheses can be made clearer and more visible. And lastly, authors can put more emphasis on significance and potential contribution of the study.

Author’s response: We have highlighted the significance and potential contribution of the review in the concluding parts of the introduction section. In addition, the specific research questions have been reduced from 5 to 2 for better clarity. (The changes can be found on page 1, 4-5).

#PRISMA
The study has largely followed the PRISMA diagram, however some important observations are as follows:

1. Provide more detail on inclusion and exclusion criteria so that reader can quickly understand the selection process (focus on exclusion criteria).

Author’s response: the inclusion criteria has been presented in the first table (Table 1), including the specific criterion and separate tables for the included and excluded information (page 7)

2. Indicate the number of duplicates removed and prominently display in the PRISMA diagram.

Author’s response: the number of duplicates removed have been stated in the text (page 8) under data extraction, as well as in Figure 1.

3. Distinguish the studies excluded during the title/abstract screening and full text screening. This clarification can clarify the review process.

Author’s response: the information has been provided in the text (page 8) under data extraction, as well as in Figure 1.

4. Provide a detail list of reasons for exclusion (I don’t mind if you put it in the Appendix). And briefly discuss this in the main body so the reader can understand the selection process.

Author’s response: The information has been tabulated for better clarity and outlined in the text as recommended (page 6-7).

5. Were other methods such as citation tracking and expert suggestion/consultation used as sources? If not please mention it briefly in the text.

Author’s response: citation tracking was performed as mentioned in the revised text (page 9) under data extraction section, as well as in Figure 1.

#Search Criteria
The database search method used in this paper seems thorough and methodological, however a few points can be clarified further.
1. Why additional databases such as Google Scholar (very popular these days) were not included in the searching process.

Author’s response: the justification for not using Google Scholar has been provided in the text (page 6).

2. What was the logical reason for not including the gray literature?

Author’s response: the justification has been provided in the revised manuscript (page 7) and further discussed in the limitation section (page 32).

3. The study mentions the usage of combination of words but does not specify the usage of Boolean operators such as (AND, OR, NOT) in their search strategy. Please provide a documentation for these operators.

Author’s response: all the search strings including the Boolean operators are provided in the Appendix (supporting file-S2-search terms and strategy).

4. The inclusion criteria were that studies in English language were include. Why other studies in regional languages were not used? As we may miss out on a big body of literature by excluding those studies. Does workplace bullying only happens in English speaking countries or where English is understood? Please provide a logical reasoning for such a decision.

Author’s response: the reasons for excluding articles written in non-English languages have been presented in the revised eligbility section (page 7). The primary reason for excluding non-English language literature is that English-language publications have extensive coverage in the global academic community, with most significant research findings published in English. Thus, focusing on English-language literature ensures access to high-quality and relevant research materials. Additionally, due to resource and time constraints, focusing on English-language literature enhances the efficiency of screening and analysis, while mitigating the complexity and risk of errors associated with translating and comprehending non-English language sources.

#Discussion
The discussion could benefit from more integration with the broader literature on workplace stress and family dynamics. While the study focuses on workplace bullying, connecting findings to related research on general workplace stress and its family impacts would provide a richer context.

Author’s response: the discussion section has been revamped and a broader perspective has been provided to discuss the findings, as well as integrating the theories identified in the review (page 22).

The authors should discuss the methodological limitation as well.

Author’s response: the methodological limitations have been discussed in the revised manuscript (page 33).

The generalizability issue should be addressed more prominently.

Author’s response: the generalizability issue has been discussed in the revised manuscript (page 33-34).

The discussion could benefit from a more detailed exploration of the practical implications of the findings.

Author’s response: we have dedicated a new section for the practical implications of the research findings (page 29-31).

This could include specific recommendations for organizations, policymakers, and practitioners on how to mitigate the negative impacts of workplace bullying on family functioning.

Author’s response: this suggestion has been effected in the practical implications section (page 29-31).

From where is am looking at the discussion section it seems dense and somewhat crowded.

Author’s response: the discussion section has been revamped and most of the results section have been incorporated into the discussion section. (page 22-29).

Perhaps breaking it down into subsection can enhance the readability and comprehension (summary of the findings, methodological limitation, theoretical implication, practical implication etc.) Over all I enjoyed reading this well written paper.

Author’s response: the suggestion has been considered in the revised manuscript.

REVIWER 2

Additional to the current discussion section, this reviewer suggests, also to widen the scope of the context of the current work. For example the authors could point out the possible effects of pre-existing psychological trauma of workers or employers/supervisors, which can jeopardize these individuals’ resources, e.g., in dealing with adverse workplace experiences.

Author’s response: the suggestion has been incorporated into the revised manuscript (page 31).

Also, although this review indicates several work domains, differences between professional domains, e.g., the domain of medicine or law, in which professional identity (professional ‘ego’) might be relevant to point out as well.

Author’s response: thanks for the suggestion; however we did not come across such event or variable in the reviewed studies.

(PS - re-reading the manuscript could help solve some last very minor punctuation issues).
Author’s response: the manuscript has been proofread thoroughly to correct typographical errors.

REVIEWER 3

Reviewer #3: I enjoyed reading this interesting systematic literature review paper.
The process of identifying papers for reveiw seems systematic and follows PRISMA framework. The writing is clear and flowing well. The structure and headings of the paper seem appropriate. However, my concern is that the theoretical contribution of the paper does not seem to be clear and strong. The authors seem to have described how many studies supported specific relationships between variables of interest. The authors should focus more on the underlying theory being tested and studeid in each paper, and provide theoretical implications emanating from the revie of these studies.

Author’s response: thanks for your suggestion, all the theories tested in the reviewed studies have been incorporated into the discussion section (Page 22-29) and a new section has been developed to cover the theoretical implications(Page 31-32).

We have made every effort to improve the manuscript and sincerely appreciate the Editors’ and Reviewers’ dedicated work. We hope the revisions meet your approval. Should you have any further questions, please feel free to contact us.

Once again, thank you very much for your comments and suggestions!

Yours sincerely

Daniella Mokhtar

daniellamokhtar@ukm.edu.my

on behalf of all the co-authors
